# Effectiveness of family support health education intervention to improve health-related quality of life among pulmonary tuberculosis patients in Melaka, Malaysia

**DOI:** 10.1186/s12890-023-02440-5

**Published:** 2023-04-25

**Authors:** Syafiq Sidqi Saidi, Rosliza Abdul Manaf

**Affiliations:** 1 Ministry of Health, Putrajaya, Malaysia; 2 Department of Community Health, Faculty of Medicine and Health Sciences, Universiti Putra Malaysia, Serdang, Malaysia

**Keywords:** Pulmonary Tuberculosis, Quality of Life, HRQoL, SF-36, Family Support, Effectiveness

## Abstract

**Background:**

Pulmonary Tuberculosis (PTB) is an important public health problem in Malaysia. In this country, limited research has been carried out on the impact of the disease on the health-related quality of life (HRQoL). Family support interventions had been shown to be effective in improving the PTB treatment outcomes.

**Objectives:**

This study aims to determine the effectiveness of a newly developed Family Support Health Education (FASTEN) intervention in improving the health-related quality of life (HRQoL) among PTB patients in Melaka, as compared to the current conventional disease management.

**Materials and methods:**

A single-blinded, randomized controlled field trial study design was conducted in Melaka from September 2019 until August 2021, involving newly diagnosed PTB patients. The participants were randomized either into the intervention group (FASTEN intervention) or into the control group (conventional management). They were interviewed by using a validated questionnaire that includes the Short Form 36 Health Survey version 2 (SF-36v2), at three time points: at diagnosis, two months and six months after diagnosis. Data were analyzed using IBM SPSS Statistics for Windows version 24. The Generalized Estimating Equations (GEE) analysis was used to evaluate the effectiveness of the intervention, in terms of the HRQoL score difference between the groups, adjusted for baseline covariates.

**Results:**

The HRQoL among PTB patients was lower than the HRQoL of general Malaysian population. Among the total 88 respondents, the three lowest HRQoL domains scores at baseline were Social Functioning (SF), Role limitation due to Physical condition (RP) and Vitality (VT) with the median (IQR) scores of 27.26 (10.03), 30.21 (11.23) and 34.77 (8.92) respectively. The median (IQR) for Physical Component Score (PCS) was 43.58 (7.44) and for Mental Component Score (MCS) was 40.71 (8.77). There were significant difference in the HRQoL median scores between the intervention group compared to the control group, as the Physical Functioning (PF) (p = 0.018), RP (p < 0.001), General Health (GH) (p < 0.001), VT (p < 0.001), SF (p < 0.001), Role limitation due to Emotional condition (RE) (p < 0.001), General Mental Health (MH) (p < 0.001), and the MCS (p < 0.001).

**Conclusion:**

The FASTEN intervention is effective to improve the overall HRQoL among PTB patients, as the HRQoL scores were significantly higher in the intervention group compared to the control group who received conventional management. Therefore, it is recommended that the TB program should incorporate the involvement of family members in the patient’s management.

**Date of registration and number:**

The protocol was registered with RCT registered body on 05/12/2019 (Australian New Zealand Clinical Trial Registry – Registration Number: ACTRN12619001720101).

## Background

In 2021, 10.6 million people fell ill with Tuberculosis (TB) worldwide, with 1.6 million deaths from the disease [1]. In the same year, from the reported new cases, about 83% were Pulmonary Tuberculosis (PTB) cases [1]. The incidence rate differs widely among countries across the world. Tuberculosis is endemic in Malaysia, as the World Health Organization (WHO) ranked this country as an intermediate TB burden country, with the incidence of 50–99 cases per 100,000 populations. In 2022, there were 25,391 new cases in Malaysia with the notification rate of about 78 cases for every 100,000 populations [2]. Melaka is one of the states in Malaysia with high TB mortality rate, an increasing trend of 27% from year 2019 to year 2020, as compared with other states in the country [2]. Even though this state has only 509 new cases in 2022, the successful treatment outcome has been plateau for the past years, with slight decreasing trend [3].

Health-related Quality of Life (HRQoL) is defined as “the extent to which patient’s subjective perception of physical, mental and social wellbeing are affected on a day to day basis by a disease and its treatment [4]. Patients with chronic diseases, including TB disease, in addition to pure physical health, they also put high value on their mental and social wellbeing. Therefore, the evaluation of this parameter has become an important health outcome, and it is an area of concern for the policy makers, health care professionals and researchers [4]. The health program routinely focuses on bacteriological markers of response and on outcomes such as cure, mortality and treatment failure, with little emphasis on the quality of life among PTB patients. Most of the PTB patients distinguish themselves to be at risk of stigma-related social and economic consequences, making them feel rejected and isolated, even from their own relatives [5]. Besides the sufferings from the disease, the treatment itself may also play a role in affecting the HRQoL, as the regime involved long duration (at least six months), with multiple drugs, as these could lead to adverse reactions among the unfortunate patients.

In order to improve PTB patients’ quality of life, it is very important to ensure that they adhere and complete their treatment. Adherence to medication is the leading key to successful outcome of the disease management, as well as to break the chain of the infection transmission [6]. And to promote the adherence behavior, the patients, as well as their close relatives, should have good knowledge on the disease, which could be obtained from the health education and awareness programs. Thus, a comprehensive and effective health program must incorporates the understanding of patients’ perception with regards to the impacts of the disease on their physical, mental, emotion and social well-being.

Pulmonary tuberculosis is a social disease; it affects the patients, as well as the people surrounding them. A successful treatment includes good social support systems a PTB patient should receives from the surrounding people [7]. Social support can be defined as the “process of interaction in relationships which improves coping, esteem, belonging, and competence through actual or perceived exchanges of physical or psychosocial resources” [7]. Family members have a huge responsibility in the management of PTB patients, because of the long treatment duration. The roles of family members should be engaged through out the disease processes; from the beginning with the diagnosis of the disease, continues with the coping of the signs and symptoms, the health-seeking behavior, and towards the end, achieving a successful treatment outcome together. It had been shown that family supports are of paramount importance in the treatment adherence, quality of care, treatment completion, treatment outcomes, as well as the psychosocial well-being among the patients [8, 9]. Family members who supported the patient through the illness made the patients responded positively to those that stayed with them [10]. This is also in line with the WHO recommendation to incorporate the family support intervention in the management of TB patients, especially in the drug-resistant cases [8].

To the best of the researcher’s knowledge, no available study has explored family support health education interventions to improve the quality of life of PTB patients in Melaka. Therefore, based on the hypothesis that comprehensive family support health education interventions might improve the quality of life of PTB patients, this study proposes a newly developed intervention, a **Fa**mily **S**uppor**t** Health **E**ducatio**n (FASTEN)**. It is a family-based education intervention, guided by the Health Belief Model principles, and consists of health education related to PTB, that was given to both the patients, but with greater emphasis on the involvement of family members as well. The intervention encourages to “fasten” or to attach both the patients and their family together in the PTB management, from the beginning to the end, in order to produce a better treatment outcome.

Therefore, the objective of this study was to determine the effectiveness of the **FASTEN** intervention in improving the HRQoL among PTB patients in Melaka, as compared to the current conventional disease management.

## Methods/design

### Study settings

Melaka is one of the thirteen states in Malaysia, with a population of around 1 million in 2022, made up of approximately 3% of the country population [11], It is 1,720 km square in total area and located in southern region of the Malaysia Peninsula, next to the Straits of Melaka. The main healthcare provider is the Ministry of Health Malaysia with the majority of TB cases being treated in their facilities.

The confirmed cases of TB are advised to take their TB medication under the direct observation of the healthcare workers. They are given routine health clinic follow-ups. Patients who loss to their follow-up are traced by medical assistant and later by a district health office team. Once a PTB patient being diagnosed, then a contacts tracing was taken place. During that investigation, family members who are living with the patient were instructed to go to the health clinic for TB screening. During the TB screening, they were given only basic information on the disease. If the screening result was positive, they will be treated as a patient. If the screening result was negative, they will be follow-up for a total duration of two years with specified appointment intervals. So, there was no continuous process of ‘involving’ them in the next six months of the patient’s management. Thus, the study aimed to introduce a family-based education intervention, known as the **FASTEN** intervention as a potential mechanism to increase the treatment outcome and eventually the quality of life of the patients.

### Study design

This is an evaluation study to determine the effectiveness of the **FASTEN** intervention. The effectiveness of the intervention was assessed using an experimental study in the form of a randomized controlled trial. This study is a two armed, parallel, single-blinded, randomized controlled field trial study design. The recruitment period was from September 2019 until September 2020. The data collection period was from September 2019 until end of April 2021.

### Recruitment of study population

All Malaysian individuals with newly diagnosed PTB, available from September 2019 until September 2020 in Melaka were eligible for the study. The eligibility of a participant was based on the inclusion and exclusion criteria. All three districts in Melaka (Melaka Tengah, Alor Gajah and Jasin) were selected, in which all the health facilities that have the capability of managing PTB were included in the study.

### Inclusion and exclusion criteria of respondents

#### Inclusion criteria

All Malaysian patients, aged ≥ 18 years old, a confirmed new PTB case and were started on TB treatment were eligible. Additionally, only literate patients and who were staying with family members (spouse, children, siblings, parents) were included.

#### Exclusion criteria

Those too ill to participate, and cases of retreatment PTB patients were excluded. PTB patients who were started on treatment from private facilities, PTB patients with other comorbidities such as diabetes, HIV, heart disease, etc. were excluded from the study. A PTB patient who is a family member to another PTB patients who has already been recruited into this study was also excluded.

#### Sample size calculation

The two means for hypothesis testing formula [12] was used for estimating the sample size for this study. Using a study by Erkan Kibrisli et al. [13], the calculated sample size was 34 (at least 17 respondents in each arm). The convenience sampling technique was used to select the respondents.

### Randomization

Random allocation of PTB patients either into the intervention arm (A) or into the control arm (B) was conducted by using permuted block randomization method. Block size of four, with six possible permutations coded from 1 to 6 was used. The six possible permutations were 1 = AABB, 2 = ABAB, 3 = ABBA, 4 = BAAB, 5 = BABA, 6 = BBAA. A random number sequence was used to generate a number to select the corresponding block to determine the allocations, until the number of sample size had achieved.

### The FASTEN intervention

The intervention is categorized as a family-focus health education. The intervention module, **Fa**mily **S**uppor**t** Health **E**ducatio**n** based on Health Belief Model principles **(FASTEN)**, aims to improve PTB patients’ quality of life, with the involvement of their family members, during the course of the disease management. The strategies under the family support health education module includes; (1) A detailed health education on the disease that was given to the patient’s family member during the home visit, (2) A copy of Pamphlets about the disease were distributed to the patient’s closest family member during the home visit, and (3) Two weekly phone text reminder about the disease to the closest family member. The content of the health education module was adapted from a similar type of study entitled “Family-Based Tuberculosis Counseling Supports Directly Observed Therapy in Armenia: A Pilot Project” by Truzyan et al., 2018. A similar approach was utilized, which aimed for members from households with TB patients to assist in assuring that TB drugs are taken regularly. It was recommended that the patient’s spouse, followed by the patient’s child, and then possibly the patient’s mother were identified as possible candidates (14). However, it depends on the personal relationships and power structures within a family. Thus, for the current study, the closest family member who the TB patient is living with is identified, as well as family member who is directly involved in the patient’s care. After both of them have consented, the healthcare provider then delivered the **FASTEN** intervention module to them. Because of the stigma associated with TB, the need to protect confidentiality, and patients’ preference, the health education session was conducted in the TB patients’ home, during a period when visitors are least expected. The whole intervention duration was a six months program, in consistence with the PTB treatment period.

### Blinding and allocation concealment

Both groups received the standard care of conventional management, with the intervention group received additional FASTEN intervention. They were explained about the intervention and asked for their consent. The researcher, who was aware of the group allocation of the patients, was not involved in the data collection process. To further preserve the effect of blinding, the data collection (through questionnaires) was done by trained medical assistants, and they were also unaware of the group allocation.

### Data collection method

This study used a self-administered questionnaire, given to the patients at the chest clinic, to assess the HRQoL among PTB patients. The questionnaire covers two sections; Section A: Socio-demographic / economic factors, and Section B: Short Form 36 (SF-36) version 2 Health Survey. The questionnaire components on section A was constructed and modified based on a previous study [15]. For section B, the validated SF-36 Health Survey (version 2.0) questionnaire was used [16–18]. The questionnaires in English and Malay languages were used for this study. SF-36v2 included eight domains including physical functioning (PF), role limitation due to physical problems (RP), bodily pain (BP), general health (GH), vitality (VT), social functioning (SF), role limitation due to emotional problems (RE), and emotional well-being (MH). The score in each dimension is graded from 0 to 100, with 0 indicates the lowest, and 100 indicates the highest quality of life [19]. The questionnaire was retrieved upon completion and was checked for completeness before the respondent left.

### Statistical analysis

The software IBM SPSS Statistics for Windows version 24 was used for data analysis [20]. The scoring data for SF-36 questionnaire was obtained from Quality Metric Health Outcomes Scoring Software before further analyzed using SPSS. In the descriptive analysis, if the data was normally distributed, the frequencies, mean and standard deviation were measured, and if the data was not normally distributed, the median and interquartile range (IQR) was measured. The 95% confidence interval (95% CI) was set for means estimation, with *p*-value at 0.05 for the level of significance to reject null hypothesis. The Generalized Estimating Equations (GEE) analysis was used to evaluate the effectiveness of the intervention, in terms of the HRQoL score difference between the groups, adjusted for baseline covariates.

**Fig. 1:**
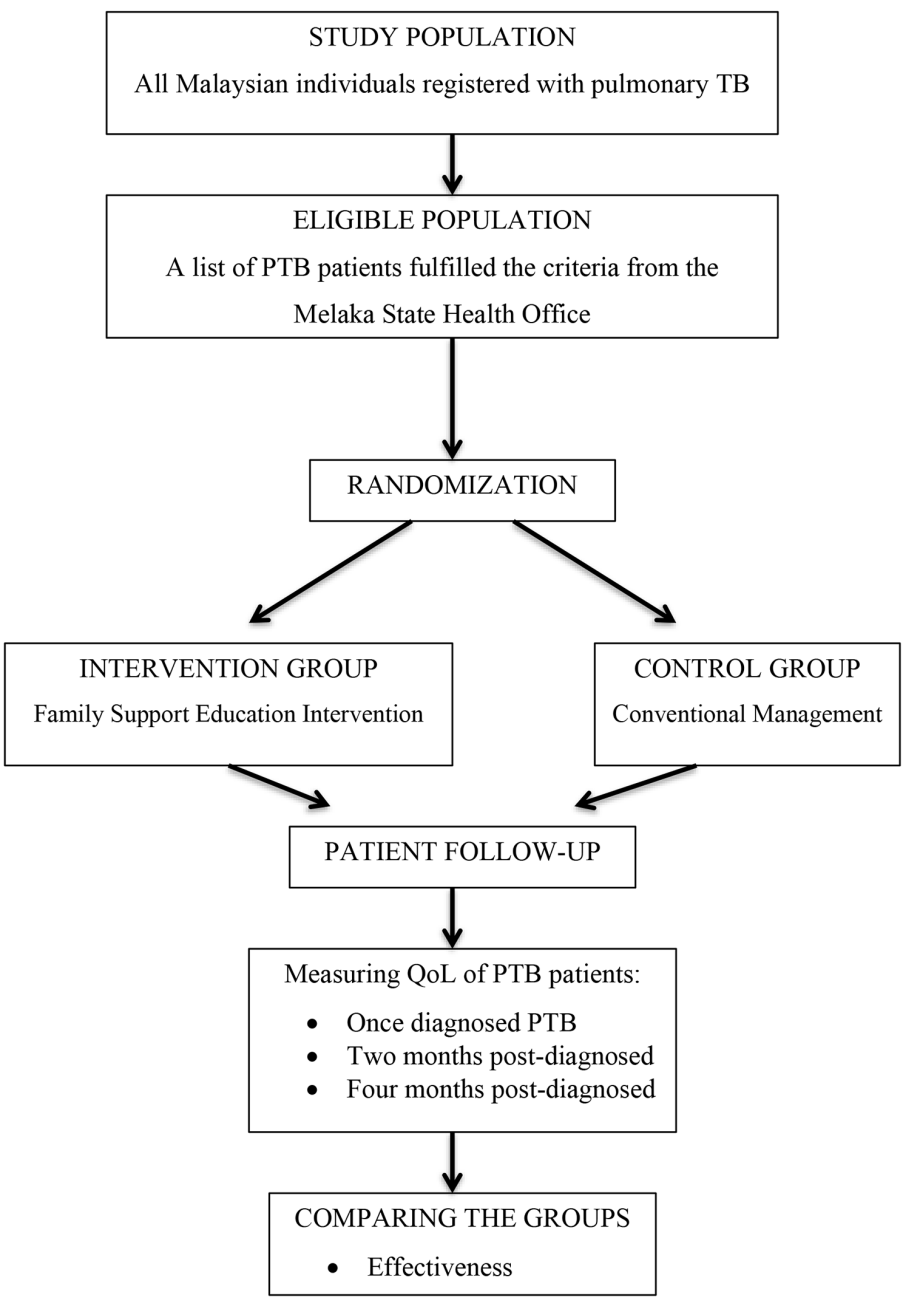
Participants’ Flow in the Study

The summary of the participants’ flow in the study is shown as in the Fig. 1.

## Results

### Respondents’ socio-demographic characteristics

A total of 88 eligible respondents were included in the study (44 in each arm). The median (IQR) age for all respondents was 38.0 (30.0) years old. Based on the Table 1, majority of the respondents came from the 18–39 years old age group (30.7%). Male patients were slightly dominant with 56.8%. Majority of the respondents interviewed were Malays (81.8%), and were Muslims (83%). The distribution of the respondents was predominantly higher from the urban residence (85.2%). Many of them were from Melaka Tengah district (64.8%), followed by Alor Gajah district (22.7%) and Jasin district (12.5%). Majority of the respondents were employed with 46.6% working as supporting staff roles and 11.4% working as professionals. With regards to the level of education, 26.1% get education from university level or higher. 69.4% of the respondents at least did their primary school, or their secondary school level of education. Majority of the respondents were married (67.0%). As for smoking habit, 52.3% were a non-smoker, 27.3% were ex-smokers and 20.5% were active smokers.

**Table 1:** Socio-demographic characteristics of the respondents (*N* = 88)

Characteristics	Total n (%)
Age (years)	
18–29	27 (30.7)
30–39	19 (21.6)
40–49	13 (14.8)
50–59	16 (18.2)
$\ge$60	13 (14.8)
Sex	
Male	50 (56.8)
Female	38 (43.2)
Race	
Malay	72 (81.8)
Chinese	10 (11.4)
Indian	5 (5.7)
Others	1 (1.1)
Religion	
Islam	73 (83)
Buddha	10 (11.4)
Hindu	5 (5.7)
Residence	
Rural	13 (14.8)
Urban	75 (85.2)
Occupation	
Unemployed	28 (31.8)
Students	9 (10.2)
Supporting staffs	41 (46.6)
Professionals	10 (11.4)
Education Level	
No education	3 (3.4)
Primary school	10 (11.4)
Secondary school	51 (58)
University or higher	23 (26.1)
Others	1 (1.1)
Marital status	
Single	27 (30.7)
Married	59 (67)
Divorced	2 (2.3)
Smoking Status	
Never	46 (52.3)
Ex-smoker	24 (27.3)
Current smoker	18 (20.5)
District	
Melaka Tengah	57 (64.8)
Alor Gajah	20 (22.7)
Jasin	11 (12.5)

### Respondents’ HRQoL scores

The median scores of the eight domains ranges from 27.26 to 62.00. The three lowest median (IQR) scores were from the SF, RP and VT domains with 27.26 (10.03), 30.21 (11.23) and 34.77 (8.92) respectively. The three highest median (IQR) scores were from the BP, GH and RE domains with 62.00 (6.45), 47.48 (12.60) and 45.72 (16.54) respectively. As for the component summaries, the median (IQR) for the Physical Component Score (PCS) was 43.58 (7.44) and for the Mental Component Score (MCS) was 40.71 (8.77), as described in Table 2.

**Table 2:** Median scores of each HRQoL domain among the respondents (*N* = 88)

SF-36v2 HRQoL Domains	Range	Median (IQR)
Physical functioning (PF)	19.26–57.54	37.45 (11.01)
Role limitation due to physical condition (RP)	21.23–57.16	30.21 (11.23)
Bodily pain (BP)	21.68–62.00	62.00 (6.45)
General health (GH)	21.33–62.70	47.48 (12.60)
Vitality (VT)	25.86–58.54	34.77 (8.92)
Social functioning (SF)	17.23–47.31	27.26 (10.03)
Role limitation due to personal emotional problem (RE)	21.35–56.17	45.72 (16.54)
General mental health (MH)	24.71–58.72	45.64 (7.85)
Physical component score (PCS)	19.06–60.86	43.58 (7.44)
Mental component score (MCS)	21.67–55.64	40.71 (8.77)

Note: PCS is summarization of PF, RP, BP and GH, MCS is summarization of VT, SF, RE and MH.

In terms of the baseline characteristic, the study indicated that there were no significant differences between the intervention group and the control group as for seven of the HRQoL domains, namely the BP (Z = -0.454, p = 0.650), the GH (F = 1.762, p = 0.745), the SF (Z = -1.225, p = 0.221), the RE (Z = -1.764, p = 0.078), the MH (Z = 0.996, p = 0.319), the MCS (F = 0.026, p = 0.114) and the PCS (F = 0.197, p = 0.251). However, there were significant differences between the intervention group and the control group as for three of the HRQoL domains, which were the PF (Z = -2.087, p = 0.037), the RP (Z -2.102, p = 0.036), and the VT (Z = -2.352, p = 0.019).

Table 3 summarized the median scores in each HRQoL domain during the three time points, and the p-values generated showed the comparison between the differences in scores by group and time. The **FASTEN** intervention does effective to improve the HRQoL among PTB patients in Melaka, and it was more effective as compared with the control group. Only the BP domain and the PCS were not showing significant results, at the end of the study. The other HRQoL domains and the MCS were significantly improved by the **FASTEN** intervention.

**Table 3:** Differences in each of HRQoL domain scores by group and time

	Intervention Group (N = 44)			Control Group (N = 44)			p-value
SF-36v2 HRQoL Domains	T_1_ Median (IQR)	T_2_ Median (IQR)	T_3_ Median (IQR)	T_1_ Median (IQR)	T_2_ Median (IQR)	T_3_ Median (IQR)	
Physical functioning (PF)	36.49 (9.57)	51.80 (7.66)	57.54 (0.00)	38.40 (14.83)	53.71 (7.66)	57.54 (0.00)	0.018*
Role limitation due to physical condition (RP)	30.21 (4.49)	40.32 (10.66)	57.16 (0.00)	30.21 (11.23)	44.81 (17.96)	54.91 (4.50)	< 0.001*
Bodily pain (BP)	62.00 (6.45)	62.00 (0.00)	62.00 (0.00)	62.00 (0.00)	62.00 (0.00)	62.00 (0.00)	0.97
General health (GH)	47.48 (13.66)	53.19 (13.67)	66.50 (1.43)	47.48 (11.53)	51.52 (9.98)	54.38 (9.51)	< 0.001*
Vitality (VT)	34.77 (8.17)	43.69 (11.89)	70.42 (5.94)	37.74 (14.86)	45.18 (14.86)	52.60 (8.91)	< 0.001*
Social functioning (SF)	27.26 (10.03)	27.26 (10.03)	57.34 (0.00)	32.27 (10.03)	37.29 (15.04)	47.31 (10.02)	< 0.001*
Role limitation due to personal emotional problem (RE)	35.28 (13.05)	45.72 (20.89)	56.17 (0.00)	45.72 (20.89)	47.46 (10.45)	52.69 (10.45)	< 0.001*
General mental health (MH)	44.33 (7.85)	48.25 (5.23)	63.95 (2.62)	48.25 (9.81)	48.25 (5.23)	50.87 (7.84)	< 0.001*
Physical component score (PCS)	42.39 (6.61)	53.32 (8.05)	59.91 (0.50)	44.60 (8.19)	53.96 (7.29)	59.00 (3.11)	0.07
Mental component score (MCS)	37.97 (9.00)	38.75 (8.73)	60.92 (2.15)	42.78 (10.27)	43.21 (9.21)	46.21 (9.55)	< 0.001*

Note: * indicates significant p-value < 0.05 from the comparison between intervention and control groups across the time

## Discussion

### HRQoL among PTB patients in Melaka

Tuberculosis has remained a major public health problem worldwide with increased morbidity and mortality. The results of the current study highlighted that PTB has significantly impacted most of the HRQoL domains among the patients. The same situation was found in Melaka. All of the HRQoL domain scores in PTB patients were lower than the norms of general Malaysian population [21]. Among the PTB patients, the three lowest HRQoL scores were observed for the domain of SF, RP and VT.

These results were a little bit different from some other studies, even with the same studied population; as BP domain, oppositely, was among the lowest baseline score in a study in Pakistan [22], and the SF and VT domains were among the highest baseline scores in China [8]. One of the plausible explanations for the differences is because of the different nature of the populations themselves, as they were not the same exact population, with different backgrounds, perceptions, cultures and values among them. However, this present study findings were quite similar with another study in Penang, Malaysia [17]. The similarities were probably because of the ‘same setting’ in Malaysia, so they probably more or less shared the same perceptions, culture and values, compared with the other regions of the world.

In this study, in terms of the component summaries, the median for PCS was 43.58 and for MCS was 40.71. However, the findings in Uganda found out that the PCS and MCS were very much lower [23]. The findings in a study in Canada, on the other hand, showed that both the component summaries were very much higher compared with this current study [24]. Like discussed earlier, the differences could possibly due to the differences in the severity of the disease itself, as well as because of the different ways on how different people reacted and responded towards the disease. However, the current study findings were comparable with the study in Penang, Malaysia [17].

Pulmonary tuberculosis can have an impact on social functioning (SF) due to social stigma associated with it. The results of the present study showed that social functioning domain was the mostly affected. Similar findings were reported in a study in Turkey [13] and in Penang, Malaysia [17]. Traditionally it is known that PTB patients feel that they are excluded from the population due to concerns mainly related to disease dissemination [13]. Due to that reason, most of the patients were affected, as they probably opted to stay at home or stay away from their loved ones, possible due to shame and fear. Furthermore, some studies have demonstrated that stigma occurs both among the patients, as well as among their surrounding people, making them opted for isolation [13].

As for the role limitation due to Physical condition (RP) domain, the present study findings showed that majority of the PTB patients had limitation in doing their ‘vigorous’ activities. These findings were supported by a study conducted in Pakistan, China and Penang, Malaysia [8, 17, 22]. It had been proven that the effects of the disease on the patients’ physicality are more dominant as compared with other manifestations [25]. Another possible reason in explaining the affected RP domain in PTB patients is with regards to their employment [25]. Working respondents might be affected more in terms of the RP as compared with non-working respondents, because of the ‘extra’ working roles that they have. Again, it might as well depend on the severity of the disease itself, as it might differ from one individual to another.

Vitality in PTB patients reflects the feelings of having energy as compared to fatigue. The study results demonstrated that most PTB patients felt fatigued and had less energy during the illness. The findings were in line with a study in Tehran [25]. It is self-explanatory as the decrease in vitality and fatigue are common clinical manifestation experienced by PTB patients, and thus affecting the VT domain. The more severe the disease, the more the patient will be affected. Another possible explanation to the reduced VT domain scores, from different perspective, is possibly because of the patient’s body reaction towards the treatment regime. Some patients might have the side effects of the treatment such as nausea, vomiting and loss of appetite. These negative side effects could lead to reduced body energy and vitality.

### The FASTEN intervention in improving HRQoL

Generally, the HRQoL scores had improved in late phases of the TB treatment. Many studies across the world have showed that the HRQoL among the patients improved after they successfully completed the treatment [5, 8, 13, 17, 18, 22–25]. The same findings were found in this current study. After six months of treatment, there was a significant improvement in all of the HRQoL domains (p < 0.001).

At the end of the current study, the improvements of HRQoL scores from the baseline were significantly higher in the intervention group as compared to the control group. Comparing the two groups, the three most significant improvements in terms of the HRQoL domains median scores in the intervention group were VT, MH and GH domains. The involvement of the family members in the patient management had improved the patients more, as in generally, and mentally, as well as their vitality – probably they were more motivated, happier and they felt more energetic when their loved ones took care of them, besides being actively participated in their treatment regime from minute one until completed the treatment.

The **FASTEN** intervention is a health education intervention, specifically on TB disease, with the main aim to involve the family members and relatives of the patient, actively, in the management of the PTB patients. While the HRQoL had improved among all participants after six months of treatment, those who received **FASTEN** intervention had demonstrated better improvement. The significant difference of the HRQoL scores were shown in PF, RP, GH, VT, SF, RE and MH domains, as well as the MCS. The outcome of the current study findings were similar with two randomized controlled trial studies in China, as one study used family support health education and another study used family-based comprehensive nursing intervention to improve the HRQoL among TB patients [26, 27].

### Study limitations

The findings of this study need to be interpreted with certain limitations in mind, as the study only included PTB patients. Thus, the study’s results could not be generalized for all types of TB, which is the Extra-PTB patients, Multi-drug Resistant TB (MDRTB), Latent TB or even pediatric TB cases. Even though PTB patients with underlying comorbidities are important group of TB patients, they were excluded from the study, as their HRQoL might already being affected before the diagnosis of TB. The sample size was small and sample population was also from a region only. Secondly, it was not blinded so the patients knew who was receiving which intervention. Thirdly, there may have been some “contamination” between the groups, with the same healthcare providers taking care of both of them. Other limitation was the possibility of the reporting bias. Since the HRQoL is a subjective perception of each individual, and it is a proxy-parameter of health outcome influenced by many factors, which in the end could leads to this type of bias. Irrespective of the limitation, this study provides early information that was lacking before in Melaka state, Malaysia.

## Conclusion

Pulmonary tuberculosis does have an effect on the patients’ HRQoL. The disease affected the patients in all eight of the HRQoL domains, with the Social Functioning, the Role limitation due to Physical condition and the Vitality domains were mostly affected. This study showed that those who received **FASTEN** intervention had demonstrated better improvement as compared with the control group, highlighted the importance of family support in the treatment of PTB patients.

## Data Availability

The datasets used and analysed during the current study available from the corresponding author on reasonable request.
